# Prenatal Exposure to Valproic Acid Across Various Indications for Use

**DOI:** 10.1001/jamanetworkopen.2024.12680

**Published:** 2024-05-22

**Authors:** Nicole E. Smolinski, Amir Sarayani, Thuy N. Thai, Sebastian Jugl, Celeste L. Y. Ewig, Almut G. Winterstein

**Affiliations:** 1 Department of Pharmaceutical Outcomes and Policy, College of Pharmacy, University of Florida, Gainesville; 2 Center for Drug Evaluation and Safety, University of Florida, Gainesville; 3 Now with Janssen Research & Development, LLC, Raritan, New Jersey; 4 Faculty of Pharmacy, HUTECH University (Ho Chi Minh City University of Technology), Ho Chi Minh City, Vietnam; 5 Department of Epidemiology, College of Medicine and College of Public Health and Health Professions, University of Florida, Gainesville

## Abstract

**Importance:**

Teratogenic outcomes associated with valproic acid use represent a substantial concern for persons of childbearing age. Regulatory agencies worldwide have enhanced warnings or implemented risk minimization programs to reduce exposure during pregnancy.

**Objectives:**

To determine pregnancy rates during valproic acid use and concomitant contraception use across indications.

**Design, Setting, and Participants:**

This retrospective cohort study used data from the Merative MarketScan commercial claims databases from January 1, 2005, to December 31, 2020, to identify female patients aged 12 to 44 years who initiated valproic acid treatment and had continuous insurance enrollment 6 months before initiation and 9 months after treatment end. A treatment episode included consecutive prescription fills that occurred within 7 days from the end of the days’ supply of the previous dispensing. Data were analyzed from March 1 to September 10, 2023.

**Main Outcomes and Measures:**

Treatment episodes were categorized by inferred indication using diagnoses preceding treatment initiation, including epilepsy, migraine or headache, mood disorders, and unknown or off-label uses. Pregnancy incidence rate ratios (IRRs) were calculated and were adjusted for age and calendar year. Contraceptive use (prescription contraceptives, intrauterine devices, and implants) during treatment was examined.

**Results:**

The cohort included 165 772 valproic acid treatment episodes among 69 390 women (mean [SD] age, 29.8 [10.0] years). Mood disorders (42.5%) were the most common indication, followed by migraine or headache (20.1%), with epilepsy playing a minor role (14.9%). Pregnancy incidence rates during valproic acid use remained unchanged, with a rate of 1.74 (95% CI, 1.14-2.53) per 100 person-years in 2005 and a rate of 1.90 (95% CI, 1.16-3.12) per 100 person-years in 2019. Compared with epilepsy, pregnancy rates were more than double for mood disorder (IRR, 2.16 [95% CI, 1.93-2.42]) and migraine or headache (IRR, 2.01 [95% CI, 1.92-2.09]). Few treatment episodes coincided with contraceptive use (37 012 [22.3%]), and oral dosage forms were the most common (27 069 [73.1%]).

**Conclusions and Relevance:**

In this cohort study of patients of childbearing age who used valproic acid, pregnancy rates during valproic acid use did not decrease despite enhanced US Food and Drug Administration safety communications, and contraception use remained low. Patients with migraine and mood disorders accounted for the largest proportion of valproic acid use and had the highest pregnancy rates, while patients with epilepsy had the lowest. These findings suggest a need to enhance efforts to mitigate prenatal exposure to valproic acid, especially for indications where the risk of use during pregnancy outweighs the benefit.

## Key Points

**Question:**

Are US Food and Drug Administration warnings to prevent prenatal exposure to valproic acid associated with changes in pregnancy risk and contraceptive use across indications?

**Findings:**

In this cohort study of 165 772 valproic acid treatment episodes among 69 390 women, pregnancy rates during treatment remained unchanged during the 15-year study and were more than doubled among users with mood disorder or migraine compared with epilepsy. Contraception use among users was uncommon, with only 22.3% of treatment episodes having a 1-day overlap of valproic acid and contraception use.

**Meaning:**

These findings suggest a need to review efforts to mitigate prenatal exposure to valproic acid, especially for clinical indications where risk of use during pregnancy outweighs therapeutic benefit and safer alternatives are available.

## Teaser

This cohort study evaluates the risk for pregnancy onset during valproic acid treatment across indications and assesses the use of contraception during treatment among US women of childbearing age.

## Introduction

Since its approval for the treatment of seizure disorders decades ago, the clinical indications for valproic acid have evolved to include migraine prophylaxis, treatment of bipolar disorder, and many off-label uses. Along with the expanding use, increasing evidence has demonstrated fetal risk.^1^ Compared with other antiepileptic medications, valproic acid carries the highest risk of any malformation, with estimates ranging from a 2- to 7-fold increase.^2,3,4,5,6,7^ Beyond malformations, prenatal exposure has been linked to neurodevelopmental disorders and decreased cognitive abilities, including lower IQ scores.^8,9,10^

In the US, prevention of prenatal exposure to valproic acid relies on a black box warning and a medication guide. The US Food and Drug Administration (FDA) has also issued multiple warnings, drug safety communications, and label changes related to valproic acid use during pregnancy and childbearing age. These warnings emphasize the risk of neural tube defects,^11^ cognitive impairment,^12^ and hearing loss.^13^ In the most recent regulatory action, a contraindication was added to the label for use during pregnancy for migraine prophylaxis. In a 2013 Drug Safety Communication, the FDA had stated valproic acid should only be used during pregnancy for epilepsy and bipolar disorder if there is no other viable medication for a patient.^14^

The European Medicines Agency has issued contraindications for use of valproic acid during pregnancy for both migraine and bipolar disorder and recommends its use during pregnancy for epilepsy only if there is no other viable treatment.^15^ To further prevent exposure, a pregnancy prevention program was implemented in 2018, including visual warnings of pregnancy risks on drug packaging and a patient alert card provided at dispensing.^15^ Such risk mitigation strategies are not indication specific, although the adherence to safe use behaviors and risk for unintended pregnancies may vary across the patient populations who represent each indication. Similarly, the World Health Organization has recommended against the use of valproic acid in women of childbearing potential and recommended the use of contraception if valproic acid is used.^16,17^

Several studies in the US and Europe^18,19,20,21,22^ have shown a decreasing trend in valproic acid use among persons of childbearing potential after the implementation of enhanced risk minimization efforts. In recent work, Al-Bahou et al^23^ noted that the rate of decrease appears to vary across indications for valproic acid use. However, whether these indication-specific differences also translate into differences in pregnancy prevention or risk for unintended pregnancy is unclear. Therefore, this study evaluated the risk for pregnancy onset during valproic acid treatment across indications and assessed the use of contraception during treatment.

## Methods

### Data Source and Study Population

We conducted a retrospective cohort study using Merative MarketScan commercial claims databases from January 1, 2005, to December 31, 2020. MarketScan contains longitudinal health care data for a nationwide sample of privately insured patients in the US, including insurance claims for inpatient and outpatient visits as well as outpatient pharmacy claims for dispensed prescriptions. Pharmacy claims data offer details on days of supply, strength, quantity, and route of administration. This study was considered exempt from review and informed consent by the University of Florida Institutional Review Board due to the use of deidentified data. Race and ethnicity data are not collected in MarketScan. We followed the Strengthening the Reporting of Observational Studies in Epidemiology (STROBE) reporting guideline.

We identified female patients aged 12 to 44 years with valproic acid prescription fills between January 1, 2005, and December 31, 2019. We excluded any invalid valproic acid pharmacy claims (ie, negative days’ supply or price). Since patients are unlikely to receive a prescription with a supply greater than 180 days, we truncated the days’ supply to the median of 37 days for any dispensing greater than 180 days. Treatment episodes were constructed by combining subsequent dispensing, allowing up to 7-day gaps between the end of the dispensed days’ supply of a fill and the dispensing date of the subsequent fill. We required treatment episodes to be at least 30 days long. Women could contribute multiple treatment episodes if all other inclusion criteria were met. To ascertain the likely indication for valproic acid use, we required 6 months of continuous enrollment for medical and prescription benefits prior to the start of each treatment episode. Moreover, we required continuous enrollment through 9 months after the end of each treatment episode to ascertain pregnancy occurrence.

### Indication Determination

Probable indications for valproic acid use were based on *International Classification of Diseases* diagnosis codes recorded on outpatient visits in the 6 months prior to the treatment episode. We grouped these codes into 4 categories: epilepsy (defined based on Clinical Classifications Software [CCS] category 83), headaches (including migraine; CCS category 84), mood disorders (CCS category 657), and unknown.^24^ The unknown classification was used if a patient did not have a corresponding diagnosis for any labeled indication categories. For patients with diagnoses in multiple categories, we assigned the indication based on the diagnosis closest to treatment episode start, resulting in mutually exclusive indication groups.

### Pregnancy Determination

We used a previously developed algorithm to identify pregnancy episodes, which Sarayani et al^25,26,27^ have successfully used in other evaluations of prenatal exposure. This algorithm is based on validated measures of pregnancy outcomes (also providing the date of pregnancy end) and gestational age to determine conception date, resulting in timed pregnancy episodes.^28,29,30^ We considered pregnancies ending in live births, stillbirths, spontaneous abortions, induced abortions, and ectopic pregnancies and those with only prenatal care visits where the outcome could not be determined. We considered an incident pregnancy relevant to our study as a pregnancy episode identified by the algorithm, where the conception date overlapped with a valproic acid treatment episode (eFigure in Supplement 1). We required conception to occur by December 2019, but the end of pregnancy could occur in 2020 for some pregnancies. Because women might have discontinued valproic acid use owing to pregnancy intent, resulting in residual drug supply that was not used, we also determined the number of pregnancies that overlapped with valproic acid use and had a new prescription fill after estimated conception in a sensitivity analysis.

### Contraceptive Use

To assess contraceptive use, we used a previously developed algorithm that determines start and stop dates for insurance-reimbursed contraception methods. This includes copper intrauterine devices (IUDs) and hormonal contraceptives in the following categories: oral, patch, vaginal ring, injectable, IUD, and implant. We captured all pharmacy dispensing and medical encounters indicating insertion or injection between the 6-month look-back period prior to the start of a valproic acid treatment episode and the end of the treatment episode. We then used the end of the dispensed days’ supply, end of contraception based on the label (eg, 3 months for injectable contraception), and removal dates for IUDs and implants to construct contraception exposure periods. If a patient switched between different contraception types, we assigned them to a multiple contraception type category. We used 2 different approaches to quantify contraception use during valproic acid treatment. First, we determined the percentage of patients who had at least 1 day of contraceptive use overlapping with a valproic acid treatment episode. The second approach assessed the proportion of valproic acid exposure days within a treatment episode that were covered by contraception. Since IUDs can be used for over 3 years, we conducted a sensitivity analysis requiring 3 years of continuous health plan coverage prior to a valproic acid treatment episode to assess contraception exposure.

### Statistical Analysis

Data were analyzed from March 1 to September 10, 2023. We estimated annual pregnancy incidence rates and contraception use prevalence during valproic acid treatment episodes and corresponding 95% CIs using the Wilson interval. For temporal trends, valproic acid treatment episodes were anchored to a calendar year based on the start date of the treatment episode.

We calculated crude and adjusted pregnancy incidence rates, incidence rate ratios (IRRs), and associated 95% CIs for each indication using a generalized estimating equation with a Poisson distribution and log-link model. In the adjusted model, we adjusted for age and calendar year. To account for the different age distributions among different indications for valproic acid and to facilitate direct comparisons with national pregnancy rates in the general population, we calculated direct age-standardized pregnancy incidence rates using 2015 US Census data for female individuals aged 12 to 44 years.^31^

Finally, we calculated the mean number of days identified pregnancies were exposed to valproic acid. All analyses and data management were completed using SAS, version 9.4 (SAS Institute Inc). Figures were constructed using R, version 4.2.1 (R Project for Statistical Computing). Two-sided *P* < .05 indicated statistical significance.

## Results

We identified 165 772 valproic acid treatment episodes among 69 390 women of childbearing age (Table 1). Most treatment episodes were associated with diagnoses of mood disorders (70 376 [42.5%]) and migraine (32 303 [19.5%]). Epilepsy contributed the smallest proportion of treatment episodes (24 638 [14.9%]). A significant portion of treatment episodes were classified as unknown conditions (38 455 [23.2%]). Age varied across indication subgroups, with epilepsy treatment episodes attributed to patients who were commonly younger (aged 12-24 years, 12 475 [50.6%]) compared with migraine treatment users, who were predominately older (aged 35-44 years, 16 267 [50.4%]). The mean (SD) duration of treatment episodes was 84.7 (134.3) days in the overall cohort, with epilepsy treatment episodes having a slightly longer duration (106.0 [189.7] days) compared with other subgroups.

**Table 1.** Baseline Characteristics of Patients With Valproic Acid Treatment Episodes by Indication

Characteristic	Indication^a^				
	All (N = 165 772)	Epilepsy (n = 24 638)	Migraine or headache (n = 32 303)	Mood disorders (n = 70 376)	Unknown (n = 38 455)
Treatment episode length, mean (SD), d	84.7 (134.3)	106.0 (189.7)	72.9 (104.2)	84.1 (124.2)	81.4 (13.0)
Age, mean (SD), y	29.8 (10.0)	26.3 (10.3)	32.2 (9.2)	29.9 (9.7)	29.8 (10.1)
Age group, y					
12-24	59 631 (36.0)	12 475 (50.6)	7836 (24.3)	25 206 (35.8)	14 114 (36.7)
25-29	16 181 (9.8)	2354 (9.6)	3237 (10.0)	6996 (9.9)	3594 (9.3)
30-34	22 334 (13.5)	2701 (11.0)	4963 (22.2)	9837 (14.0)	4833 (12.6)
35-44	67 626 (40.8)	7108 (28.8)	16 267 (50.4)	28 337 (40.3)	15 914 (41.4)
US region					
Northeast	23 004 (13.9)	3617 (14.7)	3949 (12.2)	10 476 (14.9)	4962 (12.9)
North central	39 866 (24.0)	5842 (23.7)	7962 (24.6)	16 972 (24.1)	9090 (23.6)
South	72 864 (44.0)	10 848 (44.0)	15 477 (47.9)	29 500 (41.9)	17 069 (44.4)
West	27 994 (16.9)	3970 (16.1)	4552 (14.1)	12 630 (17.9)	6842 (17.8)
Unknown	2044 (1.2)	361 (1.5)	393 (1.2)	798 (1.1)	492 (1.3)
Insurance plan type					
Preferred provider organization	97 168 (58.6)	14 670 (59.5)	19 347 (59.9)	41 223 (58.6)	21 928 (57.0)
Health maintenance organization	28 530 (17.2)	3880 (15.8)	5139 (15.9)	11 889 (16.9)	7622 (19.8)
Other	40 074 (24.2)	6088 (247) (23.5)	7817 (24.2)	17 264 (24.5)	8905 (23.2)

^a^ Unless otherwise indicated, data are expressed as No. (%) of episodes.

**Figure 1.**
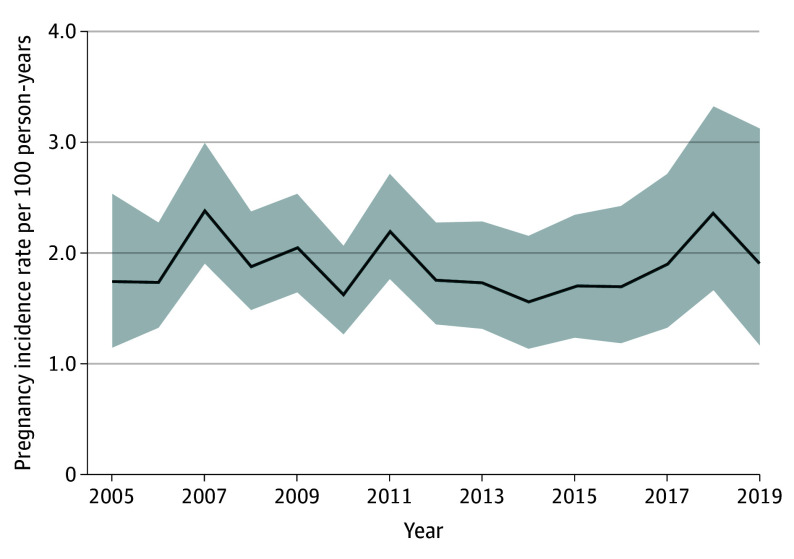
Pregnancy Incidence Rate Among Female Patients Using Valproic Acid The shaded area indicates 95% CIs.

We identified 723 pregnancies in our 165 772 treatment episodes (Table 2). The mean (SD) number of days of exposure to valproic acid during pregnancy varied across indications, with the highest among those with epilepsy (45.7 [37.1] days) followed by unknown (30.4 [32.9] days) (eTable in Supplement 1). Annual pregnancy incidence rates among all valproic acid users were steady across the study period from 1.74 (95% CI, 1.14-2.53) per 100 person-years in 2005 to 1.90 (95% CI, 1.16-3.12) per 100 person-years in 2019 (*P* = .77) (Figure 1). Crude pregnancy incidence rates differed by indication ranging from 1.22 (95% CI, 0.99-1.50) for epilepsy to 2.32 (95% CI, 2.10-2.57) for mood disorders. After age standardization, valproic acid users with migraine or headache had the highest pregnancy incidence rate of 2.70 (95% CI, 2.36-3.04), followed by those with mood disorders (2.00 [95% CI, 1.95-2.05]) and epilepsy (1.42 [95% CI, 1.10-1.74]) (Table 2). Adjusting for age and calendar year of valproic acid initiation, the pregnancy IRR for women who used valproic acid for migraine or headache was 2.01 (95% CI, 1.92-2.09) when compared with use for epilepsy. The corresponding IRR for mood disorders was 2.16 (95% CI, 1.93-2.42).

**Table 2.** Crude and Age-Standardized Pregnancy Incidence Rates Among Female Patients Using Valproic Acid by Indication

Indication	No. of episodes	No. of pregnancies	Follow-up time, d	Crude incidence rate per 100 patient-years (95% CI)	Direct age-standardized incidence rate (95% CI)	Crude IRR (95% CI)	Adjusted IRR (95% CI)^a^
All	165 772	723	14 039 505	1.88 (1.75-2.02)	2.24 (2.21-2.26)	NA	NA
Epilepsy	24 638	88	2 635 788	1.22 (0.99-1.50)	1.42 (1.10-1.74)	1 [Reference]	1 [Reference]
Migraine or headache	32 303	129	2 355 888	1.99 (1.68-2.38)	2.70 (2.36-3.04)	1.64 (1.58-1.70)	2.01 (1.92-2.09)
Mood disorders	70 376	376	5 918 214	2.32 (2.10-2.57)	2.00 (1.95-2.05)	1.90 (1.71-2.12)	2.16 (1.93-2.42)
Unknown	38 455	130	3 129 616	1.52 (1.28-1.80)	1.83 (1.61-2.05)	1.24 (1.20-1.29)	1.38 (1.33-1.44)

Abbreviations: IRR, incidence rate ratio; NA, not applicable.

^a^ Adjusted for age and calendar year.

Overall, only 445 pregnancies (61.5%) ended in a live birth (eTable in Supplement 1). Use with migraine or headache (87 of 129 [67.4%]) and unknown indication (83 of 130 [63.8%]) had a higher proportion of live births compared with those with epilepsy (49 of 88 [55.7%]) and mood disorders (226 of 376 [60.1%]). Unknown pregnancy outcomes across indications were rare, ranging from 3.1% to 6.8%.

**Figure 2.**
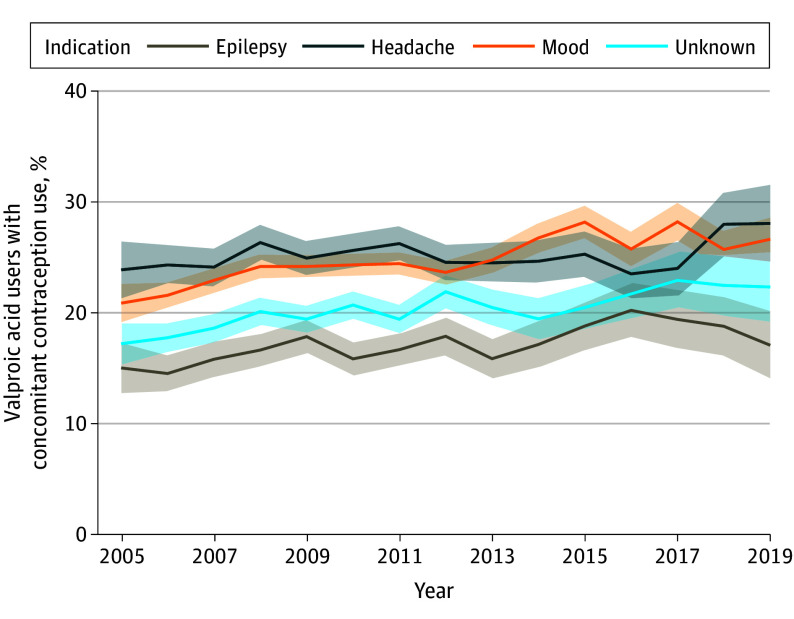
Contraception Use Among Female Patients Using Valproic Acid by Indication Contraception use is defined as at least 1 day overlap with a valproic acid treatment episode.

Limited contraceptive use was identified among valproic acid users, with 128 760 treatment episodes (77.7%) having no overlap with contraception use periods and 37 012 (22.3%) having at least 1 day of contraception coverage. Most contraception coverage consisted of oral forms (27 069 [73.1%]). Overall, there was a slight increase in identified contraception use with at least 1 day of overlap with a treatment episode from 2005 to 2019 (1167 [19.3%] to 999 [24.5%]; *P* < .001) (Figure 2). Among episodes that overlapped with contraceptive use, the most commonly identified contraceptive method included oral contraceptives (27 069 treatment episodes [16.3%]) followed by injectable contraception (4717 [2.8%]) and IUDs (1498 [0.9%]). Use of multiple contraception types accounted for 3314 episodes (2.0%). Contraception use varied slightly by indication, with higher percentages of contraceptive use among treatment episodes attributed to migraine or headache (8056 [24.9%]) and mood disorder (17 182 [24.4%]) (Table 3) when requiring at least 1 day overlap of contraception and valproic acid use. Among those using oral contraception, a median of 35.5% of the valproic acid treatment episodes was covered by contraception. This median varied slightly by indication. Median concomitant coverage was higher (100%) for users of IUDs. Overall, regardless of route or indication, there was limited contraceptive use that could be captured via pharmacy prescription fills or billed medical procedures.

**Table 3.** Contraception Use by Indication Among Female Patients Using Valproic Acid

Contraception method	Indication				
	All (N = 165 772)	Epilepsy (n = 24 638)	Migraine or headache (n = 32 303)	Mood disorders (n = 70 376)	Unknown (n = 38 455)
None	128 760 (77.7)	20 485 (83.1)	24 247 (75.1)	53 194 (75.6)	30 834 (80.2)
**Oral**					
Overlap with valproic acid use ≥1 d, No. (%)	27 069 (16.3)	2811 (11.4)	6125 (19.0)	12 338 (17.5)	5695 (14.8)
Valproic acid exposure time with contraception, median (IQR), %	35.5 (14.3-71.4)	30.8 (12.4-65.9)	41.0 (16.1-77.4)	34.9 (13.8-71.0)	37.4 (14.4-71.0)
Overlap between valproic acid exposure and contraception, median (IQR), d	23.0 (12.0-28.0)	24.0 (13.0-28.0)	23.0 (12.0-28.0)	23.0 (12.0-28.0)	23.0 (13.0-28.0)
**Intrauterine device**					
Overlap with valproic acid use ≥1 d, No. (%)	1498 (0.9)	143 (0.6)	286 (0.9)	809 (1.1)	260 (0.7)
Valproic acid exposure time with contraception, median (IQR), %	100 (87.4-100)	100 (52.7-100)	100 (100-100)	100 (88.0-100)	100 (98.5-100)
Overlap between valproic acid exposure and contraception, median (IQR), d	31.0 (31.0-91.0)	58.0 (31.0-92.0)	31.0 (31.0-87.0)	36.5 (31.0-91.0)	31.0 (31.0-91.0)
**Injectable**					
Overlap with valproic acid use ≥1 d, No. (%)	4717 (2.8)	837 (3.4)	886 (2.7)	1985 (2.8)	1009 (2.6)
Valproic acid exposure time with contraception, median (IQR), %	67.7 (28.6-71.4)	58.1 (23.4-100)	78.0 (33.3-100)	68.7 (29.3-100)	64.5 (26.9-100)
Overlap between valproic acid exposure and contraception, median (IQR), d	31.0 (23.0-47.0)	31.0 (23.0-56.0)	31.0 (24.0-39.0)	31.0 (22.0-44.0)	31.0 (23.0-51.0)
**Implant**					
Overlap with valproic acid use ≥1 d, No. (%)	414 (0.2)	46 (0.2)	66 (0.2)	232 (0.3)	70 (0.2)
Valproic acid exposure time with contraception, median (IQR), %	100 (84.1-100)	100 (61.9-100)	100 (100-100)	100 (87.7-100)	100 (71.1-100)
Overlap between valproic acid exposure and contraception, median (IQR), d	36.0 (31.0-91.0)	53.0 (31.0-122.0)	31.0 (31.0-61.0)	53.0 (31.0-91.0)	31.0 (31.0-84.0)
**Multiple contraception types**					
Overlap with valproic acid use ≥1 d, No. (%)	3314 (2.0)	316 (0.3)	693 (2.1)	1718 (2.4)	587 (1.5)
Valproic acid exposure time with contraception, median (IQR), %	77.4 (32.3-100)	57.1 (20.3-100)	68.0 (35.3-100)	77.4 (33.1-100)	77.4 (32.3-100)
Overlap between valproic acid exposure and contraception, median (IQR), d	52.0 (30.0-62.0)	56.0 (31.5-91.0)	50.0 (28.0-62.0)	52.0 (30.0-62.0)	48.0 (28.0-60.0)

In our sensitivity analysis, we identified 200 treatment episodes (27.7%) with an overlapping pregnancy that had a second dispensing of valproic acid after the conception date. In our second sensitivity analysis, where we required 3 years of continuous insurance enrollment, we identified 57 304 valproic acid treatment episodes. The use of IUDs doubled from 0.9% in the primary analysis to 1.9%. However, overall contraception use remained low, with 14 613 patients (25.5%) having at least 1 day of contraception use during a treatment episode.

## Discussion

Our study had 4 key findings that deserve discussion. First, consistent with previous findings,^32^ epilepsy played a minor role as an indication for valproic acid use among women of childbearing age, while mood disorders were the most common indication. Second, pregnancy incidence rates varied across indications, with epilepsy having the lowest rate, even after age standardization. This implies that use of valproic acid for epilepsy has the smallest contribution to prenatal exposure and ultimately, adverse pregnancy outcomes. Pregnancy incidence rates for migraine or headache and mood disorder treatment episodes were more than double the risk identified for epilepsy treatment episodes, with both indications carrying a contraindication for use during pregnancy in the European Union and use for migraine contraindicated in the US. Third, despite several safety communications and labeling changes focused on enhanced warnings to not use valproic acid during pregnancy, pregnancy risk among users did not decline over our 15-year study period. Finally, prescription contraception use among women of childbearing age who use valproic acid improved only slightly during the study period and remained generally limited regardless of indication.

With epilepsy providing the strongest favorable benefit to risk for use during pregnancy, the discovered lower pregnancy rates may seem surprising. We speculate that because most studies that demonstrate fetal risks were conducted in patients with epilepsy and typically published in neurology-centric journals, perhaps neurologists may be more informed than other specialties about the risks. Pregnancy complications associated with epilepsy itself may also emphasize the need for careful pregnancy planning, including preemptive switches to other anticonvulsants if pregnancy is desired.

We found the highest age-standardized pregnancy rates among patients using valproic acid for migraine prophylaxis and mood disorders. Compared with epilepsy, migraine prophylaxis has multiple other viable medications other than valproic acid, resulting in agreement among the US and European regulatory agencies regarding an absolute contraindication for use during pregnancy. Although opinions diverge about the treatment of mood disorders, it would be relevant to explore whether treatment modifications were considered as part of prepregnancy care, or whether exposure was mostly accidental.

Both the FDA and European Medicines Agency recommend contraception in persons of childbearing potential using valproic acid regardless of indication.^14,15^ The Centers for Disease Control and Prevention estimates that 14.0% of women of childbearing age (15-49 years) use oral contraceptives, 10.4% use reversible long-acting contraception, and 3.1% use injections, rings, or patches.^33^ We found lower use of contraception for valproic acid users, with an overall 22.3% of treatment episodes having at least 1 day of contraception coverage. Patients with epilepsy had less contraception use, which could be due to different pregnancy prevention behaviors. However, age may play a role since patients with epilepsy are younger than patients with migraine or mood disorders. While contraception use increased over the study period to 24.5% in 2019, this proportion is likely not representative of all women in the study population who were sexually active. One study assessing contraception use among patients with epilepsy who used medications associated with teratogenic outcomes^34^ found that 26% had prescription contraception use (ie, oral contraception, IUD, or injection). While there are other contraception methods available to persons of childbearing potential that do not require a prescription or medical intervention (eg, barrier methods), they are not recommended due to lower efficacy.^35,36^

Although pregnancy rates were lower than in the general population (1.88 per 100 person-years among valproic acid users compared with 8.7), prenatal exposure to valproic acid does occur.^37^ When considering the risk and benefit of valproic acid use during pregnancy, one must consider the impact on the fetus and mother. Hence, risk mitigation considerations for valproic acid have initially weighed the risk of uncontrolled epilepsy against the teratogenic risk of valproic acid use. With the broadened indications, this equation has now been changed to include predominantly scenarios where risk of use during pregnancy outweighs benefit and hence, prenatal exposure should be avoided. This finding, coupled with the observation that although valproic acid use has decreased, pregnancy risk has not, suggests that current risk mitigation approaches should be reevaluated and place greater emphasis on prevention of prenatal exposure among persons of childbearing age who use valproic acid for migraine prophylaxis and bipolar disorder, for which alternative medications could reasonably be used. Perhaps indication-specific risk mitigation approaches focusing on migraine prophylaxis and mood disorders are needed. Such enhanced efforts to prevent pregnancy during valproic acid treatment are particularly relevant in light of recent changes in abortion laws, limiting options for pregnancy termination if unintended prenatal exposure occurs. There is a need to assess causes of such exposure to aid in the development and implementation of risk mitigation measures. Likewise, there is a need to understand barriers to the use of contraception to prevent unintended pregnancies.

### Limitations

This study has several limitations. First, our data did not provide the exact clinical indication for use. Therefore, we inferred the indications based on diagnoses from outpatient medical claims preceding treatment episodes. Given the consistency of our findings with those of the literature (eg, minimal valproic acid use for epilepsy), our approach has likely had reasonable accuracy. Second, in our main analysis, we only included contraception use indicated by claims during our 6-month look-back period, which might have missed implants and IUDs that were placed previously. In a sensitivity analysis, we included patients with 3 years of continuous enrollment prior to valproic acid treatment to assess contraception use and found similarly low rates. Nonprescription contraception accounts for approximately 14% of all contraception use.^33^ Therefore, we likely underestimated contraception use in our study, but the magnitude of these missing data may not fully justify the risk mitigation gap.

## Conclusions

In this cohort study, patients with migraine or headache and mood disorders accounted for the largest proportion of valproic users and had the highest pregnancy rates during use, while patients with epilepsy had the lowest. Despite a variety of safety communications and label changes issued by the FDA, pregnancy rates during valproic acid use did not decrease during the study period. Contraception use among valproic acid users remained low. Our findings indicate a need to enhance efforts to mitigate prenatal exposure to valproic acid, especially for indications where risk of use during pregnancy outweighs benefit.

## Notes

Supplement 1. **eFigure.** Measurement Framework for Pregnancy Occurrence During Valproic Acid Treatment Episode **eTable.** Pregnancy Outcomes by Indication Among Female Valproic Acid Users

Supplement 2.
Data Sharing Statement

## References

[1] Ornoy A. Valproic acid in pregnancy: how much are we endangering the embryo and fetus? Reprod Toxicol. 2009;28(1):1-10. doi:10.1016/j.reprotox.2009.02.014 19490988

[2] Weston J, Bromley R, Jackson CF, . Monotherapy treatment of epilepsy in pregnancy: congenital malformation outcomes in the child. Cochrane Database Syst Rev. 2016;11(11):CD010224. doi:10.1002/14651858.CD010224.pub2 27819746 PMC6465055

[3] Tanoshima M, Kobayashi T, Tanoshima R, Beyene J, Koren G, Ito S. Risks of congenital malformations in offspring exposed to valproic acid in utero: a systematic review and cumulative meta-analysis. Clin Pharmacol Ther. 2015;98(4):417-441. doi:10.1002/cpt.158 26044279

[4] Tomson T, Battino D, Bonizzoni E, ; EURAP Study Group. Comparative risk of major congenital malformations with eight different antiepileptic drugs: a prospective cohort study of the EURAP registry. Lancet Neurol. 2018;17(6):530-538. doi:10.1016/S1474-4422(18)30107-8 29680205

[5] Hernández-Díaz S, Smith CR, Shen A, ; North American AED Pregnancy Registry; North American AED Pregnancy Registry. Comparative safety of antiepileptic drugs during pregnancy. Neurology. 2012;78(21):1692-1699. doi:10.1212/WNL.0b013e3182574f39 22551726

[6] Vajda FJ, O’Brien TJ, Graham JE, Lander CM, Eadie MJ. Dose dependence of fetal malformations associated with valproate. Neurology. 2013;81(11):999-1003. doi:10.1212/WNL.0b013e3182a43e81 23911758

[7] Koren G, Nava-Ocampo AA, Moretti ME, Sussman R, Nulman I. Major malformations with valproic acid. Can Fam Physician. 2006;52(4):441-442, 444, 447. 16639967 PMC1481679

[8] Bromley RL, Calderbank R, Cheyne CP, ; UK Epilepsy and Pregnancy Register. Cognition in school-age children exposed to levetiracetam, topiramate, or sodium valproate. Neurology. 2016;87(18):1943-1953. doi:10.1212/WNL.0000000000003157 27581218

[9] Christensen J, Grønborg TK, Sørensen MJ, . Prenatal valproate exposure and risk of autism spectrum disorders and childhood autism. JAMA. 2013;309(16):1696-1703. doi:10.1001/jama.2013.2270 23613074 PMC4511955

[10] Christensen J, Pedersen L, Sun Y, Dreier JW, Brikell I, Dalsgaard S. Association of prenatal exposure to valproate and other antiepileptic drugs with risk for attention-deficit/hyperactivity disorder in offspring. JAMA Netw Open. 2019;2(1):e186606. doi:10.1001/jamanetworkopen.2018.6606 30646190 PMC6324310

[11] US Food and Drug Administration. Information for healthcare professionals: risk of neural tube birth defects following prenatal exposure to valproate. Updated August 15, 2013. Accessed February 10, 2022. https://wayback.archive-it.org/7993/20170406045008/https:/www.fda.gov/Drugs/DrugSafety/PostmarketDrugSafetyInformationforPatientsandProviders/DrugSafetyInformationforHeathcareProfessionals/ucm192649.htm

[12] US Food and Drug Administration. FDA Drug Safety communication: children born to mothers who took valproate products while pregnant may have impaired cognitive development. Updated February 8, 2018. Accessed February 10, 2022. https://www.fda.gov/drugs/drug-safety-and-availability/fda-drug-safety-communication-children-born-mothers-who-took-valproate-products-while-pregnant-may

[13] AbbVie Inc. Highlights of prescribing information for Depakene. Revised 2016. Accessed February 10, 2022. https://www.accessdata.fda.gov/drugsatfda_docs/label/2016/018081s065_018082s048lbl.pdf

[14] US Food and Drug Administration. FDA Drug Safety Communication: valproate anti-seizure products contraindicated for migraine prevention in pregnant women due to decreased IQ scores in exposed children. Updated February 26, 2016. Accessed February 10, 2022. https://www.fda.gov/drugs/drug-safety-and-availability/fda-drug-safety-communication-valproate-anti-seizure-products-contraindicated-migraine-prevention

[15] European Medicines Agency. Valproate and related substances. June 7, 2018. Accessed February 10, 2022. https://www.ema.europa.eu/en/medicines/human/referrals/valproate-related-substances-0

[16] World Health Organization. mhGAP-Intervention Guide (mhGAP-IG 2.0): addendum on the use of valproic acid (sodium valproate) in women and girls of childbearing potential. 2023. Accessed February 27, 2024. https://cdn.who.int/media/docs/default-source/brain-health/mhgap_ig_v4_0(08032022).pdf?sfvrsn=696f27df_21

[17] World Health Organization. Statement on the risks associated with use of valproic acid (sodium valproate) in women and girls of childbearing potential. May 2, 2023. Accessed February 27, 2024. https://www.who.int/news/item/02-05-2023-use-of-valproic-acid-in-women-and-girls-of-childbearing-potential

[18] Degremont A, Polard E, Kerbrat S, . Impact of recommendations on sodium valproate prescription among women with epilepsy: an interrupted time-series study. Epilepsy Behav. 2021;125:108449. doi:10.1016/j.yebeh.2021.108449 34839242

[19] Ehlken B, Nishikawa C, Kaplan S, Dresco I, Granados D, Toussi M. Effectiveness of risk minimization measures for valproate: a drug utilization study based on implementation of a risk minimization programme in Europe, analysis of data from the UK. Curr Med Res Opin. 2022;38(3):461-468. doi:10.1080/03007995.2021.1997286 34931552

[20] Toussi M, Shlaen M, Coste F, de Voogd H, Dimos V, Kaplan S. Effectiveness of risk minimisation measures for valproate: a drug utilisation study in Europe. Pharmacoepidemiol Drug Saf. 2021;30(3):292-303. doi:10.1002/pds.5166 33108674 PMC7894134

[21] Hughes JE, Buckley N, Looney Y, Curran S, Mullooly M, Bennett K. Valproate utilisation trends among women of childbearing potential in Ireland between 2014 and 2019: a drug utilisation study using interrupted time series. Pharmacoepidemiol Drug Saf. 2022;31(6):661-669. doi:10.1002/pds.5427 35285110 PMC9315025

[22] Spoendlin J, Blozik E, Graber S, . Use of valproate in pregnancy and in women of childbearing age between 2014 and 2018 in Switzerland: a retrospective analysis of Swiss healthcare claims data. Swiss Med Wkly. 2021;151:w20386. doi:10.4414/smw.2021.20386 33423241

[23] Al-Bahou J, Smolinski NE, Sarayani A, Thai T, Jugl S, Winterstein AG. (2-219) A comprehensive evaluation of valproic acid use among women of child-bearing age in the United States. Presented at the ASHP Midyear Clinical Meeting; Las Vegas, Nevada; December 5, 2022.

[24] Clinical Classifications Software (CCS) for ICD-9-CM. 2022. Accessed February 10, 2022. https://www.hcup-us.ahrq.gov/toolssoftware/ccs/ccs.jsp#download

[25] Sarayani A, Albogami Y, Elkhider M, Hincapie-Castillo JM, Brumback BA, Winterstein AG. Comparative effectiveness of risk mitigation strategies to prevent fetal exposure to mycophenolate. BMJ Qual Saf. 2020;29(8):636-644. doi:10.1136/bmjqs-2019-010098 31649165

[26] Sarayani A, Albogami Y, Thai TN, . Prenatal exposure to teratogenic medications in the era of Risk Evaluation and Mitigation Strategies. Am J Obstet Gynecol. 2022;227(2):263.e1-263.e38. doi:10.1016/j.ajog.2022.01.004 35032444

[27] Thai TN, Sarayani A, Wang X, Albogami Y, Rasmussen SA, Winterstein AG. Risk of pregnancy loss in patients exposed to mycophenolate compared to azathioprine: a retrospective cohort study. Pharmacoepidemiol Drug Saf. 2020;29(6):716-724. doi:10.1002/pds.5017 32347619

[28] Zhu Y, Hampp C, Wang X, . Validation of algorithms to estimate gestational age at birth in the Medicaid Analytic eXtract—quantifying the misclassification of maternal drug exposure during pregnancy. Pharmacoepidemiol Drug Saf. 2020;29(11):1414-1422. doi:10.1002/pds.5126 32909348

[29] Sarayani A, Wang X, Thai TN, Albogami Y, Jeon N, Winterstein AG. Impact of the transition from *ICD-9-CM* to *ICD-10-CM* on the identification of pregnancy episodes in US health insurance claims data. Clin Epidemiol. 2020;12:1129-1138. doi:10.2147/CLEP.S269400 33116906 PMC7571578

[30] Moll K, Wong HL, Fingar K, . Validating claims-based algorithms determining pregnancy outcomes and gestational age using a linked claims-electronic medical record database. Drug Saf. 2021;44(11):1151-1164. doi:10.1007/s40264-021-01113-8 34591264 PMC8481319

[31] US Census Bureau. National Population by Characteristics. 2010-2019. Revised October 8, 2021. Accessed June 21, 2022. https://www.census.gov/data/datasets/time-series/demo/popest/2010s-national-detail.html

[32] Adedinsewo DA, Thurman DJ, Luo YH, Williamson RS, Odewole OA, Oakley GP Jr. Valproate prescriptions for nonepilepsy disorders in reproductive-age women. Birth Defects Res A Clin Mol Teratol. 2013;97(6):403-408. doi:10.1002/bdra.23147 23733498

[33] Daniels K, Abma JC. Current contraceptive status among women aged 15-49: United States, 2017-2019. NCHS Data Brief. 2020;2020(388):1-8. 33151146

[34] Bhakta J, Bainbridge J, Borgelt L. Teratogenic medications and concurrent contraceptive use in women of childbearing ability with epilepsy. Epilepsy Behav. 2015;52(Pt A):212-217. doi:10.1016/j.yebeh.2015.08.004 26460786

[35] World Health Organization. Family Planning—A Global Handbook for Providers. World Health Organization; 2018.

[36] Centers for Disease Control and Prevention. US medical eligibility criteria for contraceptive use, 2016 (US-MEC). Reviewed March 27, 2023. Accessed September 10, 2023. https://www.cdc.gov/reproductivehealth/contraception/mmwr/mec/summary.html

[37] Maddow-Zimet I, Kost K. Pregnancies, births and abortions in the United States, 1973–2017: national and state trends by age. March 2021. Accessed September 29, 2023. https://www.guttmacher.org/report/pregnancies-births-abortions-in-united-states-1973-2017

