# Supplementary material for: Prenatal Exposure to Valproic Acid Across Various Indications for Use
Source: JAMA Netw Open. 2024 May 22;7(5):e2412680. doi: 10.1001/jamanetworkopen.2024.12680 (PMC11112441; doi:10.1001/jamanetworkopen.2024.12680)
Supplement: Supplement 1. — eFigure. Measurement Framework for Pregnancy Occurrence During Valproic Acid Treatment Episode eTable. Pregnancy Outcomes by Indication Among Female Valproic Acid Users [file jamanetwopen-e2412680-s001.pdf]

Supplementary Online Content

Smolinski NE, Sarayani A, Thai TN, Jugl S, Ewig CLY, Winterstein AG. Prenatal exposure to valproic acid across various indications for use. *JAMA Netw Open*. 2024;7(5):e2412680. doi:10.1001/jamanetworkopen.2024.12680

eFigure. Measurement Framework for Pregnancy Occurrence During Valproic Acid Treatment Episode

eTable. Pregnancy Outcomes by Indication Among Female Valproic Acid Users

This supplementary material has been provided by the authors to give readers additional information about their work.

© 2024 Smolinski NE et al. *JAMA Network Open*.

eFigure. Measurement Framework for Pregnancy Occurrence During Valproic Acid Treatment Episode

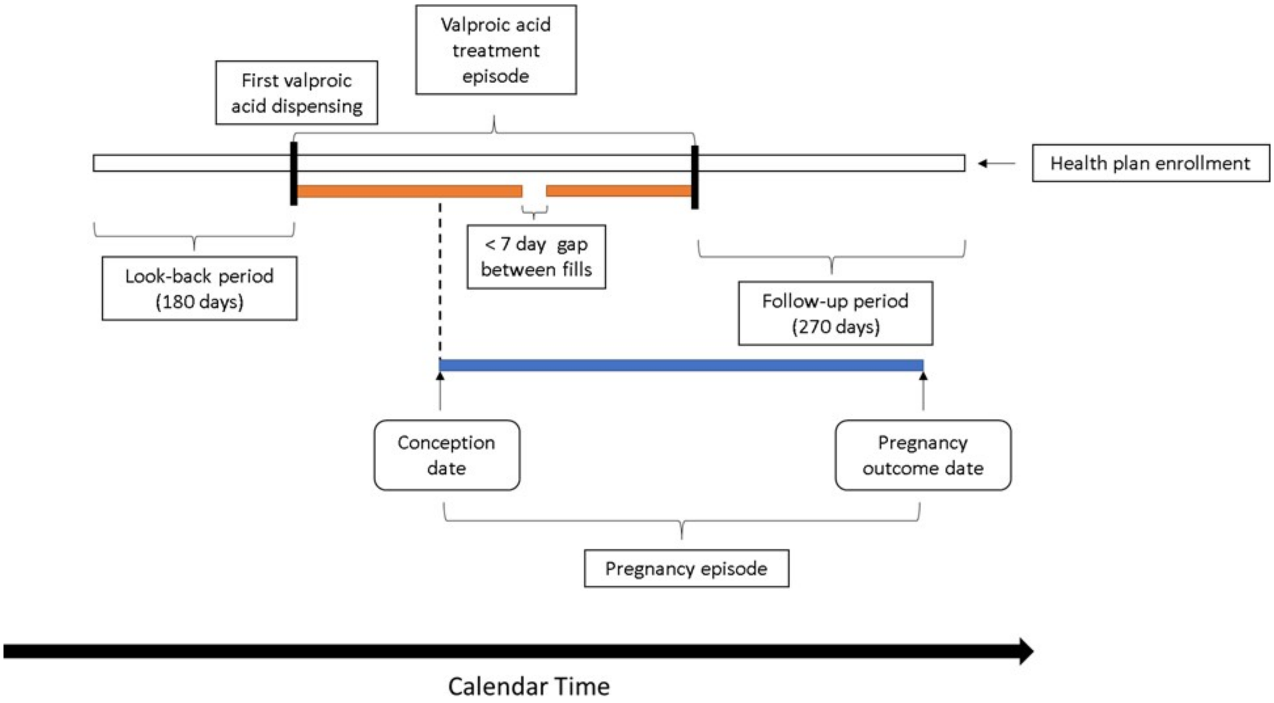

eTable. Pregnancy Outcomes by Indication Among Female Valproic Acid Users

Pregnancy Outcome	Overall N = 723	Epilepsy N = 88	Migraine/ Headache N = 129	Mood Disorders N = 376	Unknown N = 130
Livebirth	445 (61.6)	49 (55.7)	87 (67.4)	226 (60.1)	83 (63.9)
Non-Livebirths	242 (33.5)	33 (37.5)	38 (29.5)	130 (34.6)	41 (31.5)
Unknown	36 (5.0)	6 (6.8)	4 (3.1)	20 (5.3)	6 (4.6)
Average pregnancy days exposed, mean (SD)	31.0 (32.2)	45.7 (37.1)	24.6 (26.1)	30.0 (31.7)	30.4 (32.9)

© 2024 Smolinski NE et al. *JAMA Network Open*.
