# Supplementary material for: Prenatal Exposure to Valproic Acid Across Various Indications for Use
Source: JAMA Netw Open. 2024 May 22;7(5):e2412680. doi: 10.1001/jamanetworkopen.2024.12680 (PMC11112441; doi:10.1001/jamanetworkopen.2024.12680)
Supplement: Supplement 2. — Data Sharing Statement [file jamanetwopen-e2412680-s002.pdf]

Data Sharing Statement

Smolinski. Prenatal Exposure to Valproic Acid Across Various Indications for Use. *JAMA Netw Open*. Published May 22, 2024. doi:10.1001/jamanetworkopen.2024.12680

Data
Data available: No
